# Supplementary material for: Transcriptomic Insights into Mechanisms of Early Seed Maturation in the Garden Pea (Pisum sativum L.)
Source: Cells. 2020 Mar 23;9(3):779. doi: 10.3390/cells9030779 (PMC7140803; doi:10.3390/cells9030779)
Supplement: Supplementary file 1 [file cells-09-00779-s001.zip › Supplementary materials/FileS3.zip!/FileS2/Hormones - Results/Summary Sheets - Modules/Correlation Cluster/Report.pdf]

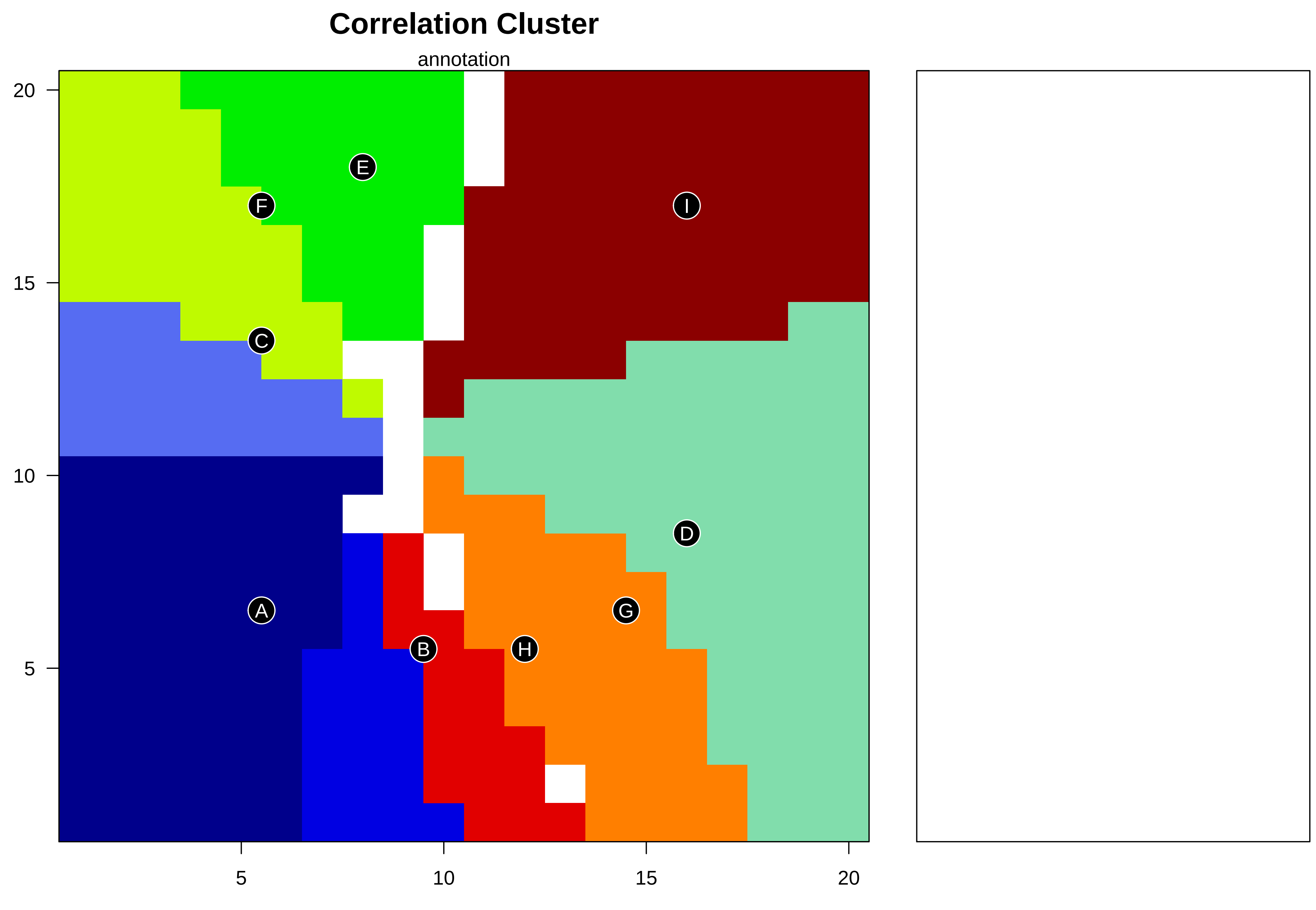
Correlation Cluster
annotation
20
15
10
5
5
10
15
20
A
B
C
D
E
F
G
H
I

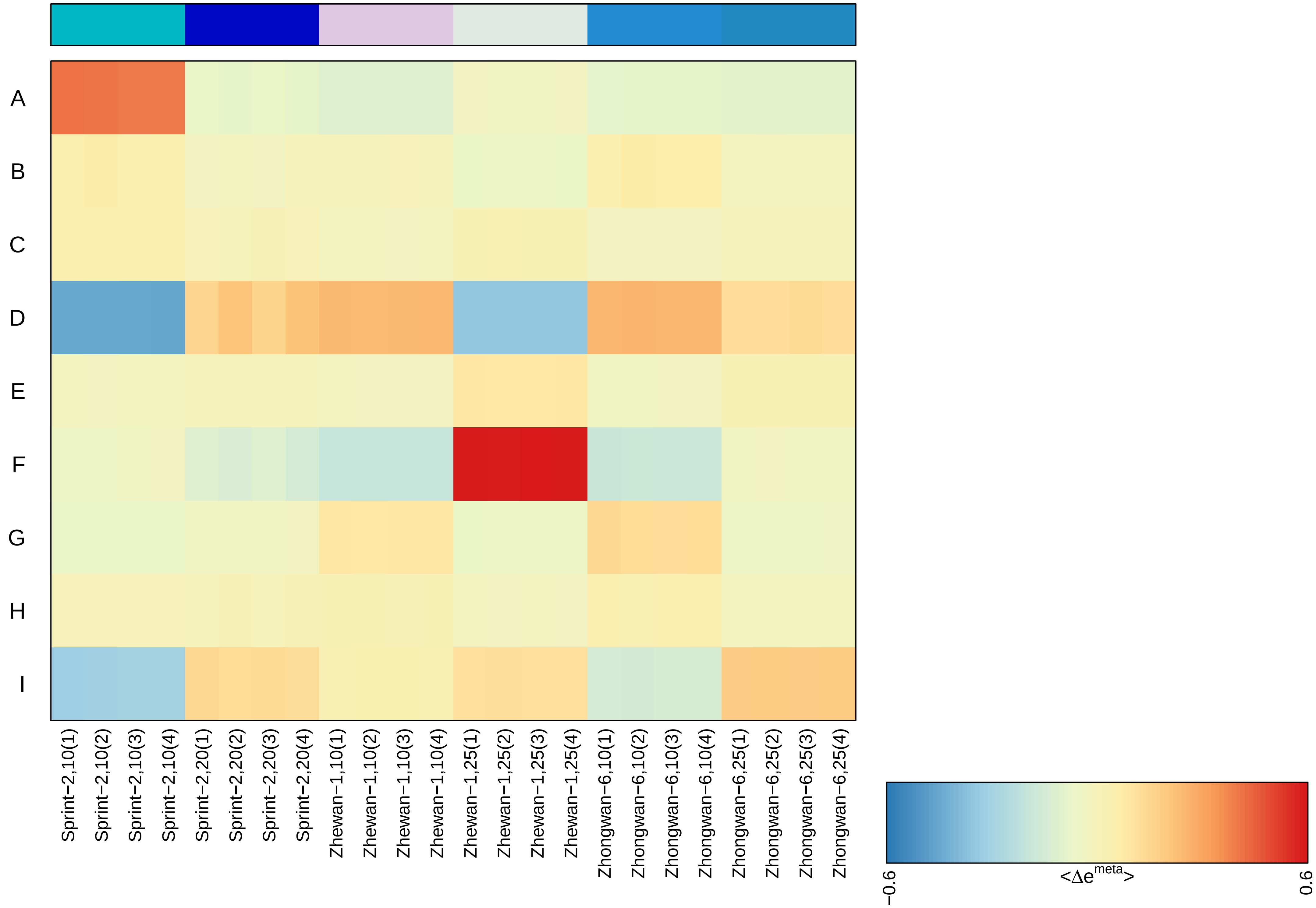

A
B
C
D
E
F
G
H
I
Sprint−2,10(1)
Sprint−2,10(2)
Sprint−2,10(3)
Sprint−2,10(4)
Sprint−2,20(1)
Sprint−2,20(2)
Sprint−2,20(3)
Sprint−2,20(4)
Zhewan−1,10(1)
Zhewan−1,10(2)
Zhewan−1,10(3)
Zhewan−1,10(4)
Zhewan−1,25(1)
Zhewan−1,25(2)
Zhewan−1,25(3)
Zhewan−1,25(4)
Zhongwan−6,10(1)
Zhongwan−6,10(2)
Zhongwan−6,10(3)
Zhongwan−6,10(4)
Zhongwan−6,25(1)
Zhongwan−6,25(2)
Zhongwan−6,25(3)
Zhongwan−6,25(4)
−0.6
<Δe^meta>
0.6

Correlation Cluster

Spot Summary: A

metagenes = 66
genes = 131

<r> metagenes = 0.95
<r> genes = 0.73
beta: r2= 0.14 / log p= −1.13

samples with spot = 4 (16.7 %)
Sprint−2(10) : 4 (100 %)

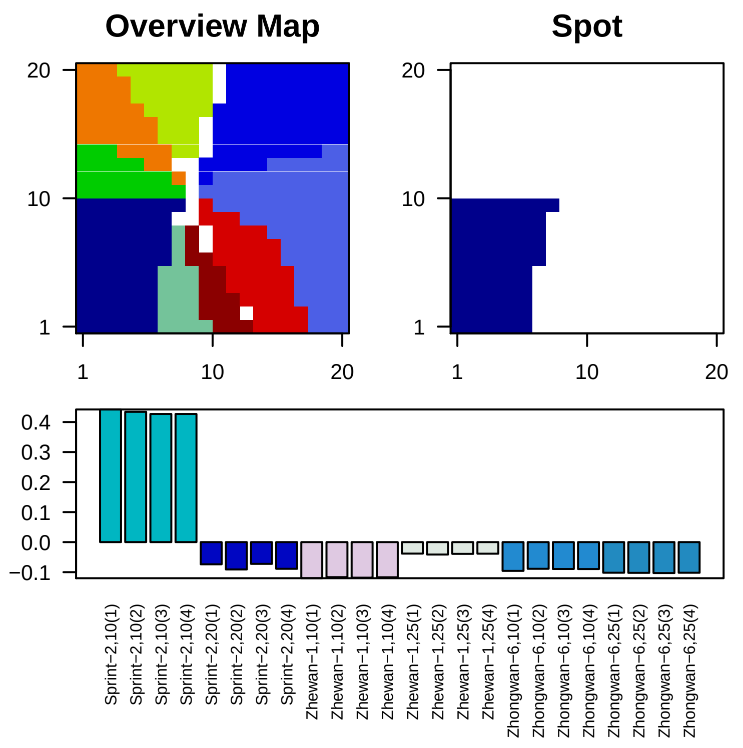

Spot Genelist

Rank	ID	max e	min e	r	Symbol	Description
1	TRINITY_DN1	15.48	−3.34	0.91		
2	TRINITY_DN3	15.03	−3.06	0.97		
3	TRINITY_DN4	4.64	−1.14	1		
4	TRINITY_DN8	3.6	−0.71	0.99		
5	TRINITY_DN4	3.56	−0.75	0.98		
6	TRINITY_DN8	3.06	−0.71	0.99		
7	TRINITY_DN6	2.31	−0.98	0.36		
8	TRINITY_DN3	1.81	−0.35	0.89		
9	TRINITY_DN3	1.66	−0.44	0.97		
10	TRINITY_DN1	1.51	−0.8	0.83		
11	TRINITY_DN5	1.42	−0.47	0.86		
12	TRINITY_DN1	1	−0.39	0.84		
13	TRINITY_DN2	0.93	−0.39	0.96		
14	TRINITY_DN1	0.85	−0.17	0.98		
15	TRINITY_DN2	0.69	−0.19	0.97		
16	TRINITY_DN8	0.62	−0.27	0.72		
17	TRINITY_DN1	0.55	−0.07	0.9		
18	TRINITY_DN1	0.54	−0.21	0.96		
19	TRINITY_DN6	0.54	−0.13	0.96		
20	TRINITY_DN6	0.48	−0.11	0.99		

Correlation Cluster

Spot Summary: B

metagenes = 19
genes = 8

<r> metagenes = 0.93
<r> genes = 0.44
beta: r2= 0.03 / log p= −0.37

samples with spot = 0 (0 %)

Spot Genelist

Rank	ID	max e	min e	r	Symbol	Description
1	TRINITY_DN1	0.3	−0.22	0.88		
2	TRINITY_DN4	0.21	−0.13	0.74		
3	TRINITY_DN4	0.18	−0.11	0.92		
4	TRINITY_DN2	0.09	−0.07	0.65		
5	TRINITY_DN4	0.07	−0.06	0.45		
6	TRINITY_DN1	0.04	−0.03	0.58		
7	TRINITY_DN3	0.04	−0.02	0.47		
8	TRINITY_DN3	0.03	−0.01	0.49		

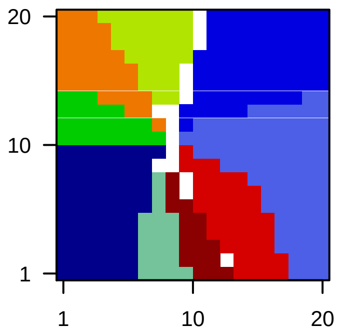

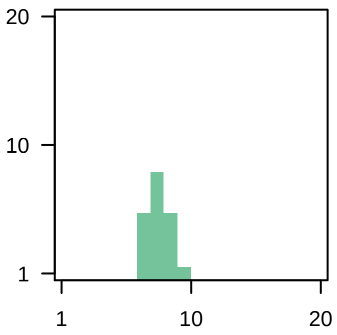

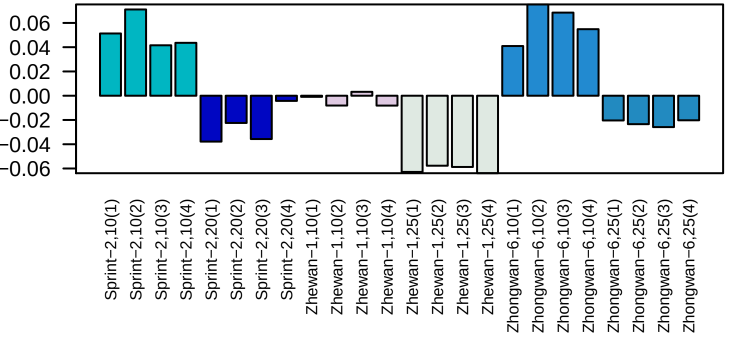

Correlation Cluster

Spot Summary: C

metagenes = 23
genes = 37

<r> metagenes = 0.97
<r> genes = 0.58
beta: r2= 0.01 / log p= −0.24

samples with spot = 0 (0 %)

Overview Map

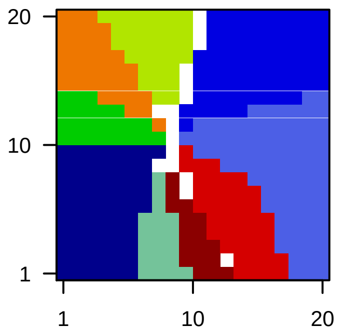

Spot

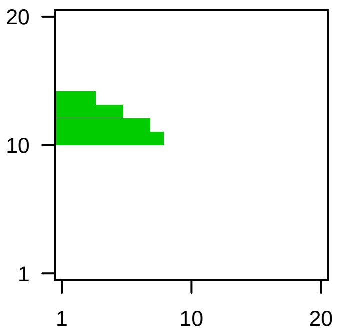

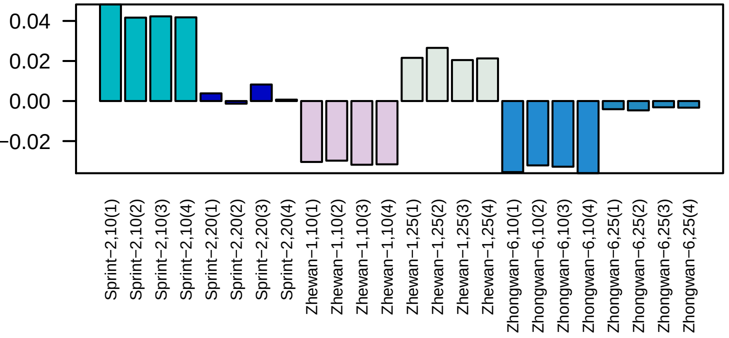

Spot Genelist

Rank	ID	max e	min e	r	Symbol	Description
1	TRINITY_DN5	0.34	−0.34	0.71		
2	TRINITY_DN6	0.29	−0.24	0.98		
3	TRINITY_DN4	0.13	−0.14	0.95		
4	TRINITY_DN7	0.1	−0.08	0.7		
5	TRINITY_DN7	0.1	−0.02	0.85		
6	TRINITY_DN7	0.09	−0.03	0.89		
7	TRINITY_DN2	0.09	−0.08	0.81		
8	TRINITY_DN4	0.09	−0.04	0.89		
9	TRINITY_DN5	0.08	−0.05	0.85		
10	TRINITY_DN5	0.07	−0.13	0.8		
11	TRINITY_DN9	0.06	−0.04	0.79		
12	TRINITY_DN1	0.06	−0.05	0.95		
13	TRINITY_DN2	0.06	−0.06	0.94		
14	TRINITY_DN8	0.06	−0.06	0.81		
15	TRINITY_DN7	0.06	−0.04	0.72		
16	TRINITY_DN1	0.05	−0.05	0.76		
17	TRINITY_DN4	0.05	−0.04	0.87		
18	TRINITY_DN9	0.05	−0.05	0.91		
19	TRINITY_DN1	0.04	−0.03	0.94		
20	TRINITY_DN6	0.04	−0.04	0.84		

Correlation Cluster

Spot Summary: D

metagenes = 81
genes = 61

<r> metagenes = 0.95
<r> genes = 0.46
beta: r2= 0.29 / log p= −2.2

samples with spot = 10 (41.7 %)
Sprint−2(20) : 2 (50 %)
Zhewan−1(10) : 4 (100 %)
Zhongwan−6(10) : 4 (100 %)

Overview Map

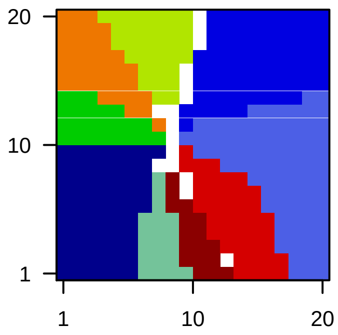

Spot

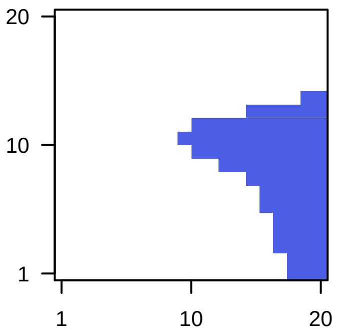

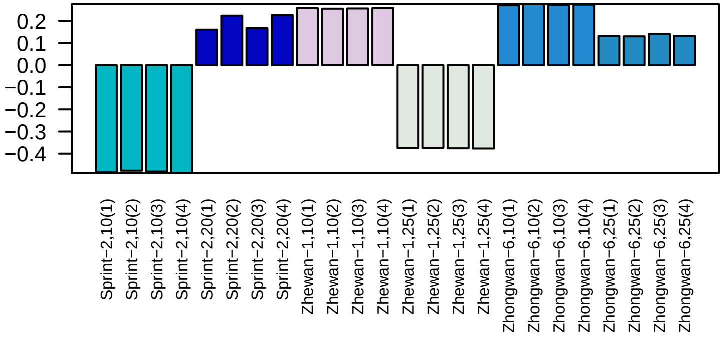

Spot Genelist

Rank	ID	max e	min e	r	Symbol	Description
1	TRINITY_DN1	9.22	−1.86	0.55		
2	TRINITY_DN3	8.41	−2.58	0.33		
3	TRINITY_DN1	5.71	−13.31	0.99		
4	TRINITY_DN5	3.72	−1.76	0.51		
5	TRINITY_DN2	2.73	−3.16	0.84		
6	TRINITY_DN4	1.96	−0.45	0.52		
7	TRINITY_DN2	1.83	−1.59	0.56		
8	TRINITY_DN3	1.75	−1.32	0.85		
9	TRINITY_DN6	1.68	−1.13	0.64		
10	TRINITY_DN3	0.64	−0.6	0.94		
11	TRINITY_DN3	0.62	−0.3	0.7		
12	TRINITY_DN1	0.61	−0.36	0.76		
13	TRINITY_DN2	0.53	−0.16	0.58		
14	comp15249_c	0.39	−0.2	0.67		
15	TRINITY_DN3	0.36	−0.11	0.54		
16	TRINITY_DN4	0.31	−0.18	0.72		
17	TRINITY_DN3	0.31	−0.25	0.77		
18	TRINITY_DN6	0.29	−0.29	0.93		
19	TRINITY_DN4	0.28	−0.24	0.86		
20	TRINITY_DN1	0.27	−0.18	0.77		

Correlation Cluster

Spot Summary: E

metagenes = 32
genes = 43

<r> metagenes = 0.93
<r> genes = 0.66
beta: r2= 0.04 / log p= −0.48

samples with spot = 0 (0 %)

Spot Genelist

Rank	ID	max e	min e	r	Symbol	Description
1	TRINITY_DN1	0.4	−0.22	0.95		
2	TRINITY_DN6	0.28	−0.1	0.95		
3	TRINITY_DN1	0.22	−0.06	0.93		
4	TRINITY_DN4	0.22	−0.09	0.97		
5	TRINITY_DN1	0.2	−0.07	0.95		
6	TRINITY_DN3	0.2	−0.1	0.89		
7	TRINITY_DN1	0.2	−0.06	0.97		
8	TRINITY_DN1	0.19	−0.11	0.97		
9	TRINITY_DN1	0.17	−0.04	0.89		
10	TRINITY_DN5	0.16	−0.09	0.85		
11	TRINITY_DN8	0.16	−0.14	0.31		
12	TRINITY_DN5	0.16	−0.13	0.9		
13	TRINITY_DN3	0.14	−0.08	0.59		
14	TRINITY_DN8	0.14	−0.14	0.75		
15	TRINITY_DN1	0.13	−0.05	0.87		
16	TRINITY_DN1	0.12	−0.09	0.23		
17	TRINITY_DN2	0.12	−0.03	0.54		
18	TRINITY_DN8	0.11	−0.11	0.87		
19	TRINITY_DN1	0.1	−0.04	0.94		
20	TRINITY_DN4	0.1	−0.02	0.65		

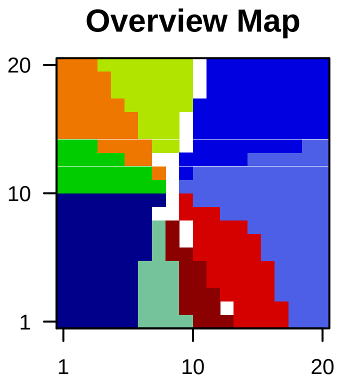

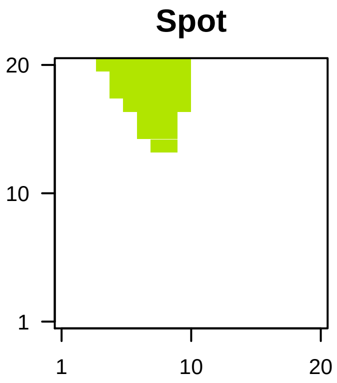

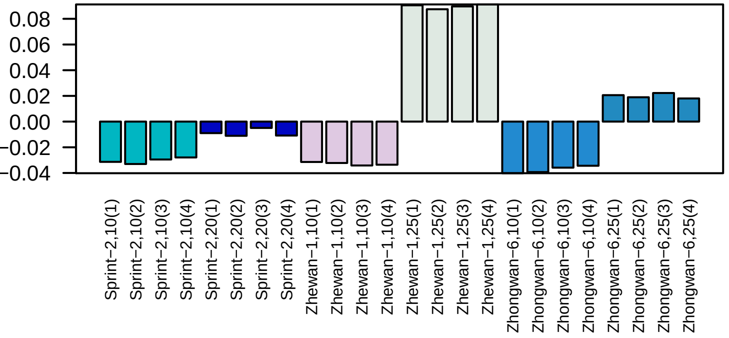

Correlation Cluster

Spot Summary: F

metagenes = 35
genes = 36

<r> metagenes = 0.97
<r> genes = 0.64
beta: r2= 0.15 / log p= −1.21

samples with spot = 4 (16.7 %)
Zhewan−1(25) : 4 (100 %)

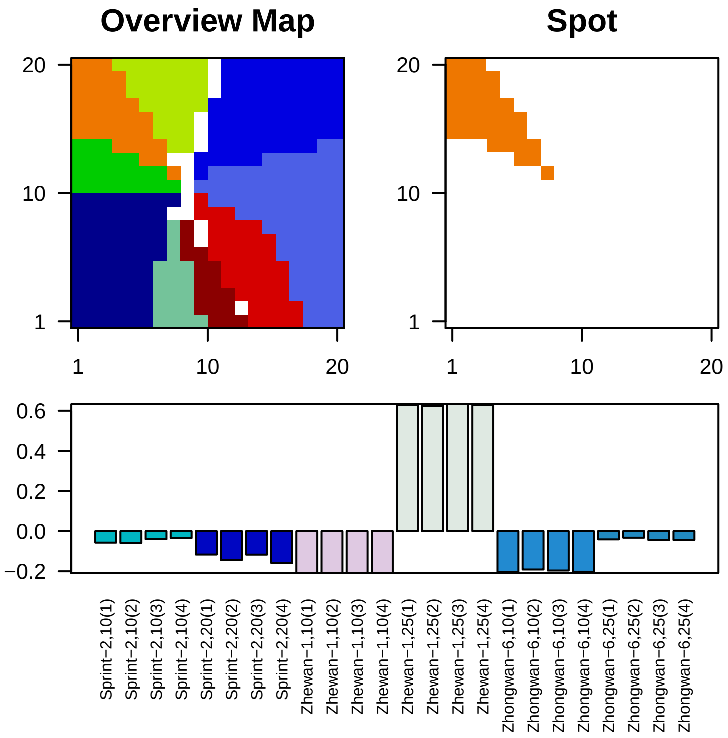

Spot Genelist

Rank	ID	max e	min e	r	Symbol	Description
1	TRINITY_DN3	15.54	−3.43	0.98		
2	TRINITY_DN1	1.68	−0.51	0.99		
3	TRINITY_DN1	0.79	−0.37	0.94		
4	TRINITY_DN5	0.75	−0.46	0.67		
5	TRINITY_DN5	0.74	−0.39	0.9		
6	TRINITY_DN4	0.59	−0.48	0.84		
7	TRINITY_DN1	0.52	−0.35	0.79		
8	TRINITY_DN5	0.47	−0.16	0.97		
9	TRINITY_DN8	0.37	−0.25	0.65		
10	TRINITY_DN3	0.29	−0.14	0.79		
11	TRINITY_DN3	0.26	−0.26	0.47		
12	TRINITY_DN5	0.21	−0.28	0.65		
13	TRINITY_DN1	0.19	−0.26	0.55		
14	TRINITY_DN4	0.17	−0.16	0.8		
15	TRINITY_DN3	0.15	−0.15	0.67		
16	TRINITY_DN6	0.12	−0.14	0.67		
17	TRINITY_DN1	0.11	−0.07	0.76		
18	TRINITY_DN3	0.11	−0.15	0.69		
19	TRINITY_DN1	0.1	−0.03	0.86		
20	TRINITY_DN4	0.07	−0.03	0.84		

Correlation Cluster

Spot Summary: G

metagenes = 40
genes = 91

<r> metagenes = 0.97
<r> genes = 0.5
beta: r2= 0.07 / log p= −0.66

samples with spot = 0 (0 %)

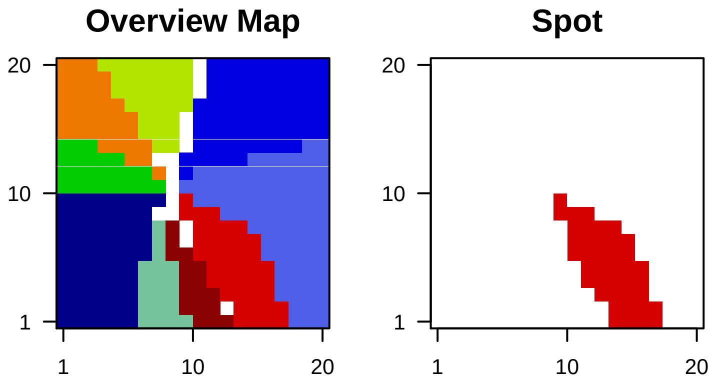

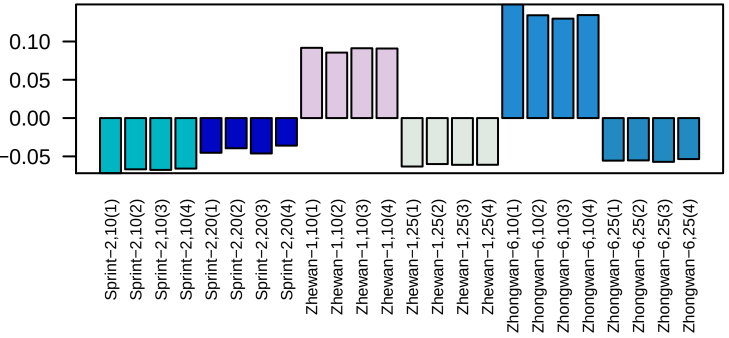

Spot Genelist

Rank	ID	max e	min e	r	Symbol	Description
1	TRINITY_DN4	1.16	−0.51	0.9		
2	TRINITY_DN6	1.09	−0.33	0.71		
3	TRINITY_DN1	1.04	−0.18	0.73		
4	TRINITY_DN1	1.03	−0.19	0.76		
5	TRINITY_DN2	1	−0.17	0.76		
6	comp40971_c	0.9	−0.39	0.85		
7	TRINITY_DN1	0.83	−0.22	0.66		
8	TRINITY_DN6	0.82	−0.22	0.67		
9	TRINITY_DN1	0.81	−0.23	0.71		
10	TRINITY_DN4	0.7	−0.29	0.99		
11	TRINITY_DN4	0.62	−0.35	0		
12	TRINITY_DN1	0.6	−0.15	0.92		
13	TRINITY_DN3	0.57	−0.12	0.84		
14	TRINITY_DN2	0.57	−0.1	0.79		
15	TRINITY_DN3	0.54	−0.17	0.62		
16	TRINITY_DN1	0.53	−0.11	0.83		
17	TRINITY_DN3	0.52	−0.14	0.87		
18	TRINITY_DN3	0.47	−0.23	0.96		
19	TRINITY_DN5	0.44	−0.12	0.56		
20	TRINITY_DN1	0.42	−0.18	0.89		

Correlation Cluster

Spot Summary: H

metagenes = 17
genes = 12

<r> metagenes = 0.95
<r> genes = 0.47
beta: r2= 0.01 / log p= −0.2

samples with spot = 0 (0 %)

Overview Map

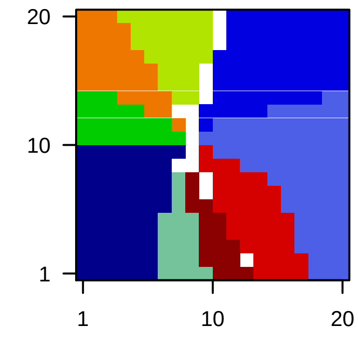

Spot

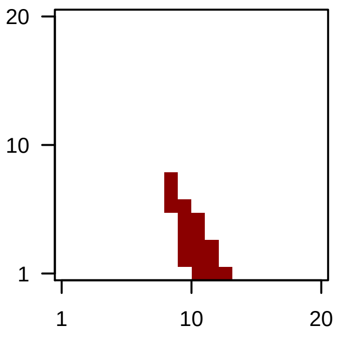

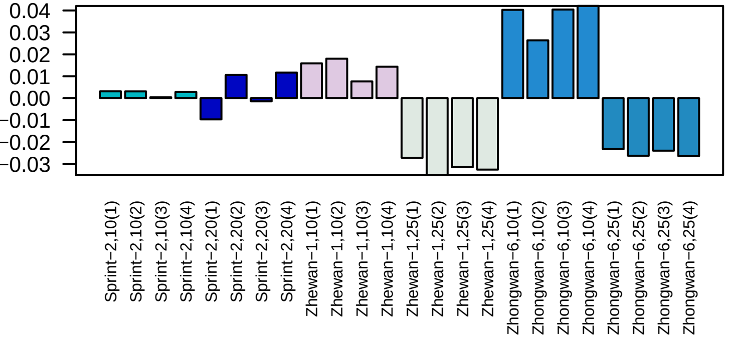

Spot Genelist

Rank	ID	max e	min e	r	Symbol	Description
1	TRINITY_DN4	0.24	−0.16	0.84		
2	TRINITY_DN1	0.15	−0.1	0.22		
3	TRINITY_DN5	0.11	−0.16	0.45		
4	TRINITY_DN1	0.1	−0.05	0.71		
5	TRINITY_DN2	0.07	−0.05	0.87		
6	TRINITY_DN9	0.06	−0.01	0.42		
7	TRINITY_DN1	0.06	−0.04	0.73		
8	TRINITY_DN3	0.05	−0.03	0.63		
9	TRINITY_DN3	0.04	−0.02	0.71		
10	comp4201_c0	0.03	−0.01	0.72		
11	TRINITY_DN8	0.03	−0.02	0.89		
12	TRINITY_DN1	0.02	−0.01	0.57		

Correlation Cluster

Spot Summary: I

metagenes = 71
genes = 59

<r> metagenes = 0.95
<r> genes = 0.42
beta: r2= 0.14 / log p= −1.15

samples with spot = 4 (16.7 %)
Zhongwan−6(25) : 4 (100 %)

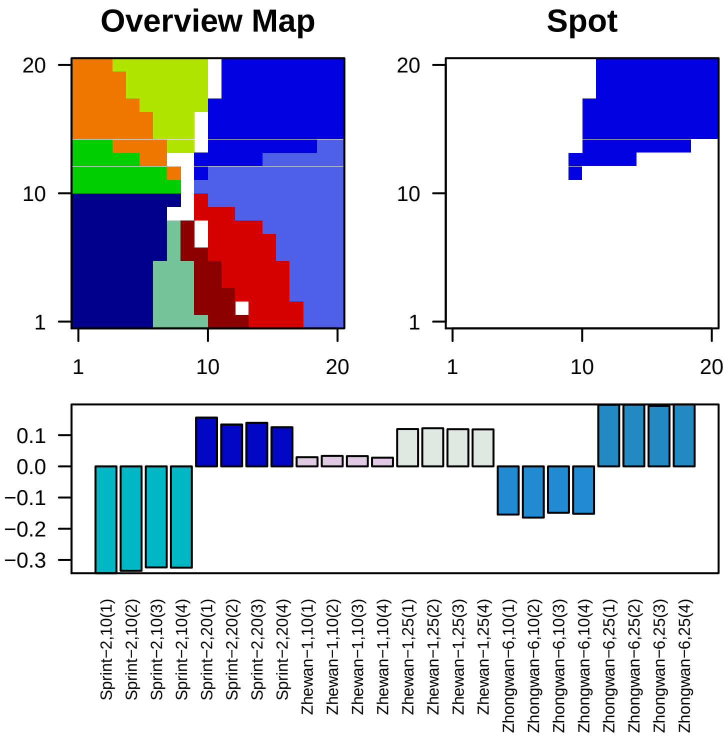

Spot Genelist

Rank	ID	max e	min e	r	Symbol	Description
1	TRINITY_DN1	3.01	−8.1	0.96		
2	TRINITY_DN3	2.24	−1.85	0.8		
3	TRINITY_DN4	1.68	−0.51	0.58		
4	TRINITY_DN6	1.58	−2.5	0.88		
5	TRINITY_DN2	1.45	−0.95	0.49		
6	comp33849_c	1.11	−0.57	0.36		
7	TRINITY_DN7	1.1	−1.67	0.61		
8	TRINITY_DN6	0.64	−0.76	0.91		
9	TRINITY_DN4	0.63	−0.35	0.78		
10	TRINITY_DN3	0.57	−0.79	0.48		
11	TRINITY_DN6	0.38	−0.27	0.78		
12	TRINITY_DN5	0.35	−0.26	0.73		
13	TRINITY_DN9	0.35	−0.08	0.5		
14	TRINITY_DN4	0.31	−0.06	0.39		
15	TRINITY_DN5	0.29	−0.43	0.94		
16	TRINITY_DN4	0.29	−0.27	0.88		
17	TRINITY_DN4	0.28	−0.15	0.57		
18	TRINITY_DN5	0.27	−0.05	0.47		
19	TRINITY_DN6	0.27	−0.09	0.46		
20	TRINITY_DN2	0.26	−0.12	0.54		
